# Artificial light at night at the terrestrial-aquatic interface: Effects on predators and fluxes of insect prey

**DOI:** 10.1371/journal.pone.0240138

**Published:** 2020-10-08

**Authors:** Elizabeth Parkinson, Justine Lawson, Scott D. Tiegs

**Affiliations:** Department of Biological Sciences, Oakland University, Rochester, Michigan, United States of America; University of Fribourg, SWITZERLAND

## Abstract

The outcomes of species interactions–such as those between predators and prey–increasingly depend on environmental conditions that are modified by human activities. Light is among the most fundamental environmental parameters, and humans have dramatically altered natural light regimes across much of the globe through the addition of artificial light at night (ALAN). The consequences for species interactions, communities and ecosystems are just beginning to be understood. Here we present findings from a replicated field experiment that simulated over-the-water lighting in the littoral zone of a small lake. We evaluated responses by emergent aquatic insects and terrestrial invertebrate communities, and riparian predators (tetragnathid spiders). On average ALAN plots had 51% more spiders than control plots that were not illuminated. Mean individual spider body mass was greater in ALAN plots relative to controls, an effect that was strongly sex-dependent; mean male body mass was 34% greater in ALAN plots while female body mass was 176% greater. The average number of prey items captured in spider webs was 139% greater on ALAN mesocosms, an effect attributed to emergent aquatic insects. Non-metric multidimensional scaling and a multiple response permutation procedure revealed significantly different invertebrate communities captured in pan traps positioned in ALAN plots and controls. Control plots had taxonomic-diversity values (as H’) that were 58% greater than ALAN plots, and communities that were 83% more-even. We attribute these differences to the aquatic family Caenidae which was the dominant family across both light treatments, but was 818% more abundant in ALAN plots. Our findings show that when ALAN is located in close proximity to freshwater it can concentrate fluxes of emergent aquatic insects, and that terrestrial predators in the littoral zone can compound this effect and intercept resource flows, preventing them from entering the terrestrial realm.

## Introduction

Seemingly separate ecosystems are in fact connected by flows of resources that can substantially contribute to the structure and functioning of the recipient ecosystems [1]. Aquatic ecosystems and their adjacent riparian zones are particularly connected by resource flows [2] and are also among the most human-impacted ecosystems on Earth [3]. Little information exists, however, on how common human activities impact cross-system resource flows and the organisms that rely on them.

Most aquatic insects are biphasic, relying on terrestrial ecosystems for reproduction and completion of their lifecycles. As a result, populations emerge from aquatic ecosystems, often in discrete pulses, and these emergence events are exploited by riparian predators including spiders [4, 5], bats [6], and birds [7, 8]. Riparian spiders, both web-building and free-living, utilize their proximity to aquatic habitats to subsidize their diets with aquatic insect prey [4, 5, 9–11] with consequences for insect mortality and reproduction [12].

Among riparian spiders, members of the family Tetragnathidae, the long-jawed orb-weavers, are especially adept at subsidizing their diets with aquatic prey [4, 5, 13–16]–up to 92% of tetragnathid body carbon can stem from aquatic sources [4, 14]. Tetragnathid spider density tracks seasonal emergence events [5] and spiders position webs where emergence is greatest [15, 17]. When webs are positioned in riparian zones these numerical responses to aquatic prey have the potential to create patches of relatively high resource exchange and can facilitate the transfer of nutrients into riparian food webs.

The outcomes of species interactions–such as those between predators and prey–depend on environmental conditions that are increasingly modified by human activities. Light is among the most fundamental environmental parameters in natural ecosystems. Natural patterns of light and darkness provide organisms with cues for migration [18], guide navigation [19, 20], influence temporal niche partitioning [21], and are closely linked with circadian functioning [22]. Many species rely on daily periods of darkness, with 30% of vertebrates and 60% of invertebrates having adaptations for nocturnal activity [23]. Humans have dramatically altered natural light regimes across much of the globe through the addition of artificial light at night (ALAN) [18, 24], yet the consequences for communities and ecosystems are just beginning to be understood.

Human population centers are often located adjacent to water, making aquatic and associated ecosystems, such as riparian zones, vulnerable to ALAN impacts. Insects may be particularly sensitive because ALAN interferes with insect navigation [25, 26] and pollination [27], interrupts dispersal of aquatic invertebrates into terrestrial systems [28, 29], disrupts mating cues [30], and alters community composition [31–35]. ALAN can also increase the presence of terrestrial invertebrate predators [31, 36], including riparian predators that subsidize their diet with aquatic prey [37].

Here we present findings from a replicated field experiment that simulated over-the-water lighting in the littoral zone of a small lake. We hypothesized that the plots exposed to experimentally added ALAN would harbor different communities of invertebrates relative to control plots that did not receive additional ALAN. Furthermore, we hypothesized that aquatic invertebrates would aggregate at sites of ALAN more than their terrestrial counterparts. Lastly, we hypothesized that predatory spiders would also aggregate at sites of ALAN, and benefit through the accrual of greater individual body mass more than spiders in plots without added ALAN. Results support these hypotheses and demonstrate how ALAN near shorelines can impact the exchange of resources from aquatic to riparian ecosystems in the form of emergent insects, and the predators that consume them.

## Methods

### Experimental design

Initially we designed an experiment to evaluate ALAN-mediated top-down effects of predatory fish (*Lepomis cyanellus*) on littoral communities and ecosystems. Experimental light treatments consisted of 2 levels (ALAN plots, and non-ALAN plots to which experimental ALAN was not added) and 2 levels of fish (the presence of fish, and a control with no fish). Each treatment had 5 replicates in a fully crossed 2x2 factorial design. These treatments (fish and ALAN; no fish and ALAN; fish and no ALAN; and no fish and no ALAN) were assigned randomly to mesocosms after installation via coin tosses. However, we were unable to maintain populations of fish in the mesocosms due to suspected predation by birds, and unknown factors. No fish effects were observed for any of the many community or ecosystem parameters we examined [38]; these include fish as a main effect, and all fish interactions. Fish as a factor were not included in any analyses presented here. Permission to conduct research involving the use of fish was granted by the Oakland University Institutional Animal Care and Use Committee (IACUC) through permit #16042.

During the experiment we began to notice that tetragnathid spiders were using the mesocosms as a structure for attaching webs, much like they were using emergent vegetation in the surrounding littoral area. We seized this opportunity to test the response of this common riparian spider taxon to ALAN by: enumerating the number of tetragnathid spiders on each mesocosm and the number of insects caught in tetragnathid webs, determining spider body mass, and sampling insect communities captured in pan traps.

### Study site

This field experiment was conducted in the littoral area of Haven Hill Lake, located in Highland State Recreation Area in White Lake, Michigan (USA) (42° 38’ 45.32” N; 83° 33 13.48”). The lake is surrounded by mixed deciduous forest and isolated from direct ALAN exposure. There is no artificial lighting immediately around the lake, but the 6,000-acre Highland State Recreation Area is surrounded by low-density human development, and skyglow is present. To distinguish the two treatments we refer to them as “ALAN” and “non-ALAN”. “Non-ALAN” should not be taken to mean that the plots were completely without exposure to ALAN given the skyglow present at the site, but rather, they did not intentionally receive additional illumination as part of our experiment. The lake has an extensive area of 0.30 m to 0.45 m deep littoral zone where our experimental mesocosms were located. Permission to conduct this experiment was provided by a permit issued by the State of Michigan Department of Natural Resources (PRD-SU-2016-015) to Elizabeth Parkinson.

### Mesocosm and ALAN installation

Mesocosms were installed at the study site during the span of 3 days in June 2016. Before installation, local macrophytes and the benthos were removed by hand in each 1 m^2^ area. Twenty experimental mesocosms consisted of 4 wooden stakes supporting a 150 mm extruded plastic mesh structure (4 walls and a bottom) measuring 1 m^2^. All mesocosms were submerged with approximately 15 cm of mesh protruding above the water’s surface. Mesocosms were installed by hammering wooden stakes into the lakebed and attaching the mesh components with cable ties. After mesocosm installation, two 3.8-liter buckets of local macrophytes and sediment were returned to each mesocosm to cover the mesh bottom.

ALAN was added 1 week after mesocosm installation and originated from solar LED warm-white “path-lights” with an output of 10 lumens each and powered by a single, solar-charged battery (Alena metal path lights, Model #91755, manufactured by Moonrays Landscape Lighting). Each ALAN mesocosm was illuminated independently by 4 lights; one path light was attached to each corner of the mesocosms at approximately 30 cm above the surface of the water. Some horizontal shielding was present on path-lights, but LED bulbs were mostly exposed on all sides, leading to some horizontal light emission. Light levels were measured at the onset of the study using the application “Light Meter” version 1.1 for iPhone 6. The light meter was positioned over the center of each mesocosm, approximately 15 cm above the water’s surface. Readings were taken at 11:30 pm on June 16^th^, 2016, with an 86% full moon. Light readings were taken 3 times at each mesocosm and averaged. Lux measurements were significantly greater in ALAN versus non-ALAN treatments (ANOVA, F_1,18_ = 453.3, p<0.0001). ALAN treatments averaged 27.3 lx and non-ALAN averaged 22.7 lx.

### Invertebrate inputs

Emergent aquatic insect and terrestrial invertebrate inputs into each mesocosm were measured overnight on August 3^rd^ using pan traps. Pan traps consisted of a plastic container (58.4 cm x 41.3 cm x 15.2 cm) with approximately 2 cm of a water/dish soap mixture covering the bottom of the pan. One pan trap was placed in each mesocosm and left floating in the mesocosms overnight. On August 4^th^ pan traps were removed and the contents were poured through a sieve to capture invertebrates, which were then stored in 70% ethanol, and transported to the Aquatic Ecology Lab at Oakland University. In the lab invertebrates were enumerated and identified to lowest practicable taxonomic unit, usually family [39, 40].

### Spider abundance and body mass and spider prey abundance

Tetragnathid spiders belonging to the genus *Tetragnatha* (probably *T*. *elongata*) were enumerated on each mesocosm biweekly on three dates: July 8, July 23, and August 4, 2016. On the final date three spiders were collected from each of the mesocosms, stored in vials, and transported to the lab on ice. In the lab spiders were classified by sex, dried in the oven at 40° C for 48 h before being cooled in a desiccator, and weighed to the nearest 0.1mg. Insects that were captured in spider webs built on mesocosms were enumerated three times, biweekly, on the same dates that spiders were enumerated.

### Statistical analysis

Response variables with multiple sampling dates (spider abundance and web-captured prey abundance) were analyzed using repeated-measures analysis of variance (rmANOVA) in package ez [41] in program R. The light treatments were treated as a fixed factor, and sampling date was treated as a random factor. Because there was an interaction between the two factors, one-way ANOVA was performed as a *post hoc* analysis of individual sampling dates for spider abundance and web-captured prey abundance in package car in program R. Non-metric multidimensional scaling (NMDS) was performed in package Vegan [42] in program R to evaluate differences in emergent and terrestrial insect communities between ALAN treatments. Multi-response permutation procedure (MRPP) was used in package Vegan to test for separation of NMDS ordinations using Bray-Curtis distances. Linear regressions were performed in program R to test for relationships between emergent and terrestrial invertebrate abundance, and spider body mass and abundance [43]. Invertebrate communities were further evaluated with the Shannon-Weiner diversity index, taxonomic richness and evenness values. Hedge’s g effect sizes were calculated separately for male and female spiders to evaluate the magnitude of ALAN effects between sexes. Two-way ANOVA was used to evaluate differences in mean spider body mass between ALAN and the sex of the spiders, in package car in program R. Data were examined and determined to be sufficiently normally distributed and homoscedastic for the parametric analyses performed. The light treatment was treated as a fixed factor, as was the sex of the spiders.

## Results

### Spider abundance and body mass

**Fig 1:**
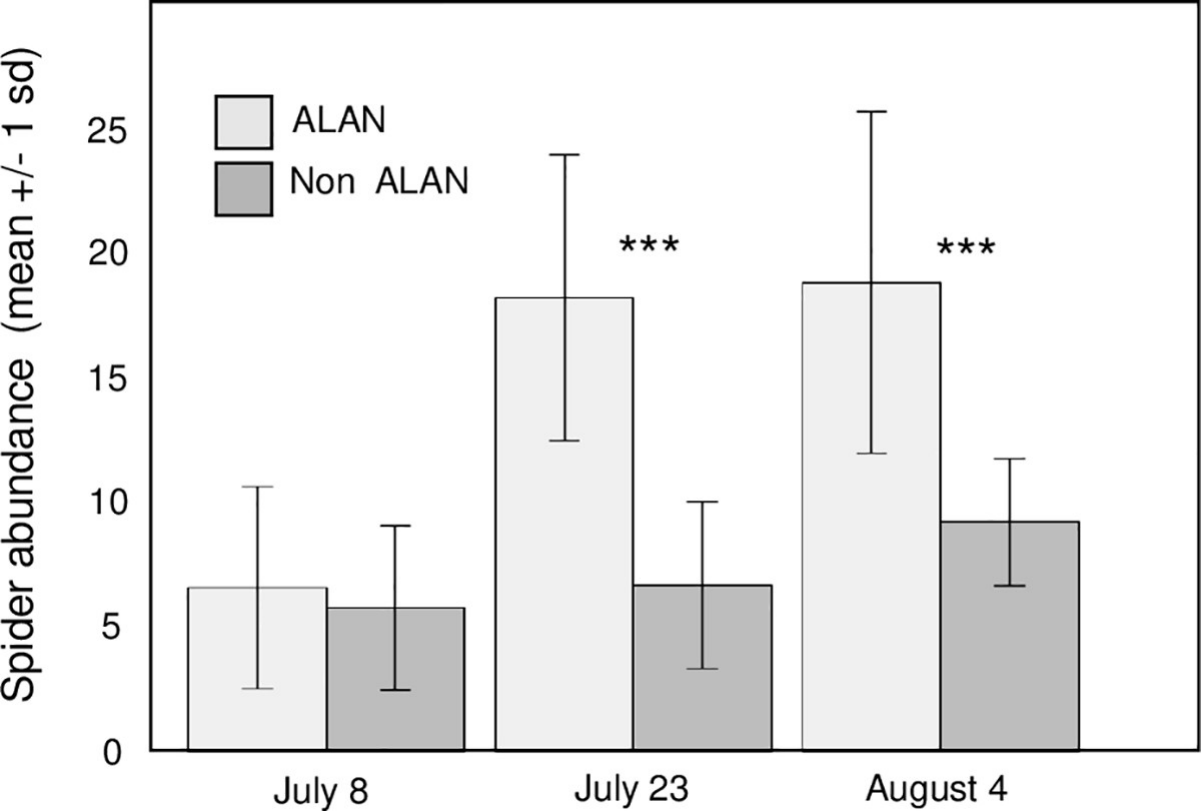
Mean number of tetragnathid spiders found per mesocosm in ALAN mesocosms versus no-ALAN mesocosms (i.e., controls) grouped by sampling date. Significantly more spiders were found in ALAN treatments (p<0.001) relative to controls, an effect that depended on sampling date. Post-hoc tests were performed on each sampling date to evaluate differences between ALAN plots and controls, and asterisks statistical significance. Error bars represent one standard deviation.

Mean spider abundance per mesocosm was greater in ALAN plots relative to non-ALAN plots. Totals of 431 and 214 spiders were enumerated on ALAN and non-ALAN plots respectively with means (±SD) of 14.37 ± 7.83 spiders in ALAN plots and 7.13 ± 3.29 spiders in non-ALAN plots. On average, across the three sampling dates, ALAN plots had 51% more spiders than plots that were not illuminated with ALAN (rmANOVA, F_1,16_ = 33.34, p<0.001) (Fig 1). The magnitude of the ALAN effect, however, depended on sampling date, evidenced by a significant interaction term between the date and the ALAN treatment (rmANOVA, F_2,36_ = 8.52, p<0.001). Post hoc tests to compare ALAN plots and controls on each individual sampling date revealed that on the first sampling date there was no ALAN effect. By the second and third date, however, the magnitude of the ALAN effect was significant and ALAN plots had 172% and 104% more spiders relative to controls.

**Fig 2:**
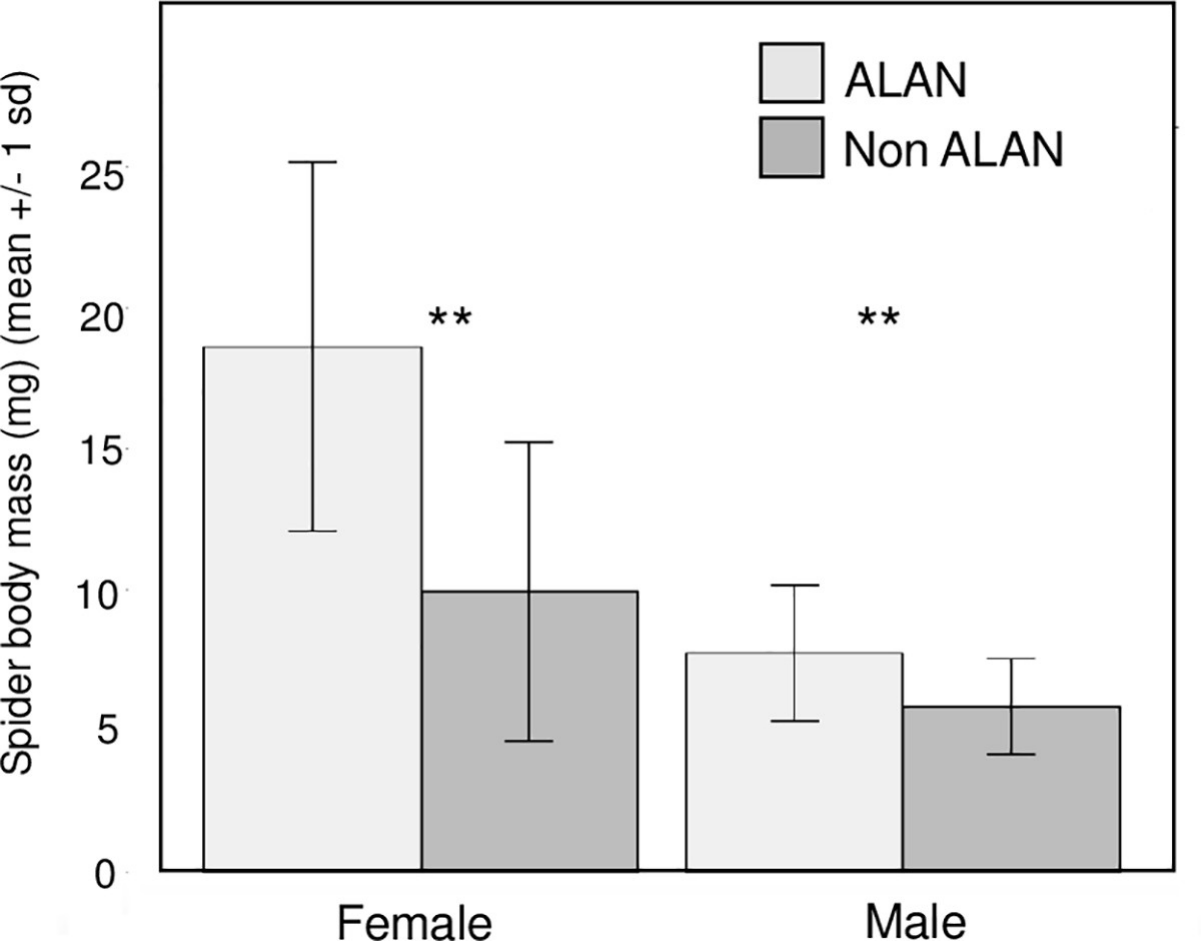
Mean body mass (+/- 1 one standard deviation) of tetragnathid spiders grouped by sex in ALAN mesocosms versus controls. Spiders on ALAN mesocosms were found to have greater body mass than those sampled on unlit plots for both sexes, and the effect was significantly greater for females; female body mass was more than two times greater in ALAN plots relative to controls.

A total of 30 males and 15 females were collected from ALAN plots and a total of 18 males and 21 females were collected from non-ALAN plots. Mean spider body mass was 101% greater in ALAN mesocosms relative to controls (ANOVA, F_1,56_ = 23.15, p<0.001), and a significant interaction was found between light treatment and spider sex (ANOVA, F_1,56_ = 6.96, p = 0.011). Post hoc tests were performed to compare ALAN effects on male and female spider body mass separately. Although body mass was greater in ALAN treatments for both sexes, the magnitude of the effect was substantially greater for females; mean male body mass was 34% greater in ALAN mesocosms relative to controls (ANOVA, F_1,46_ = 9.03, p = 0.004) while female body mass was 176% greater (ANOVA, F_1,34_ = 19.32, p<0.001) (Fig 2). These differences between sexes translate to Hedge’s g effect sizes of 1.49 for females and 0.90 for males.

**Fig 3:**
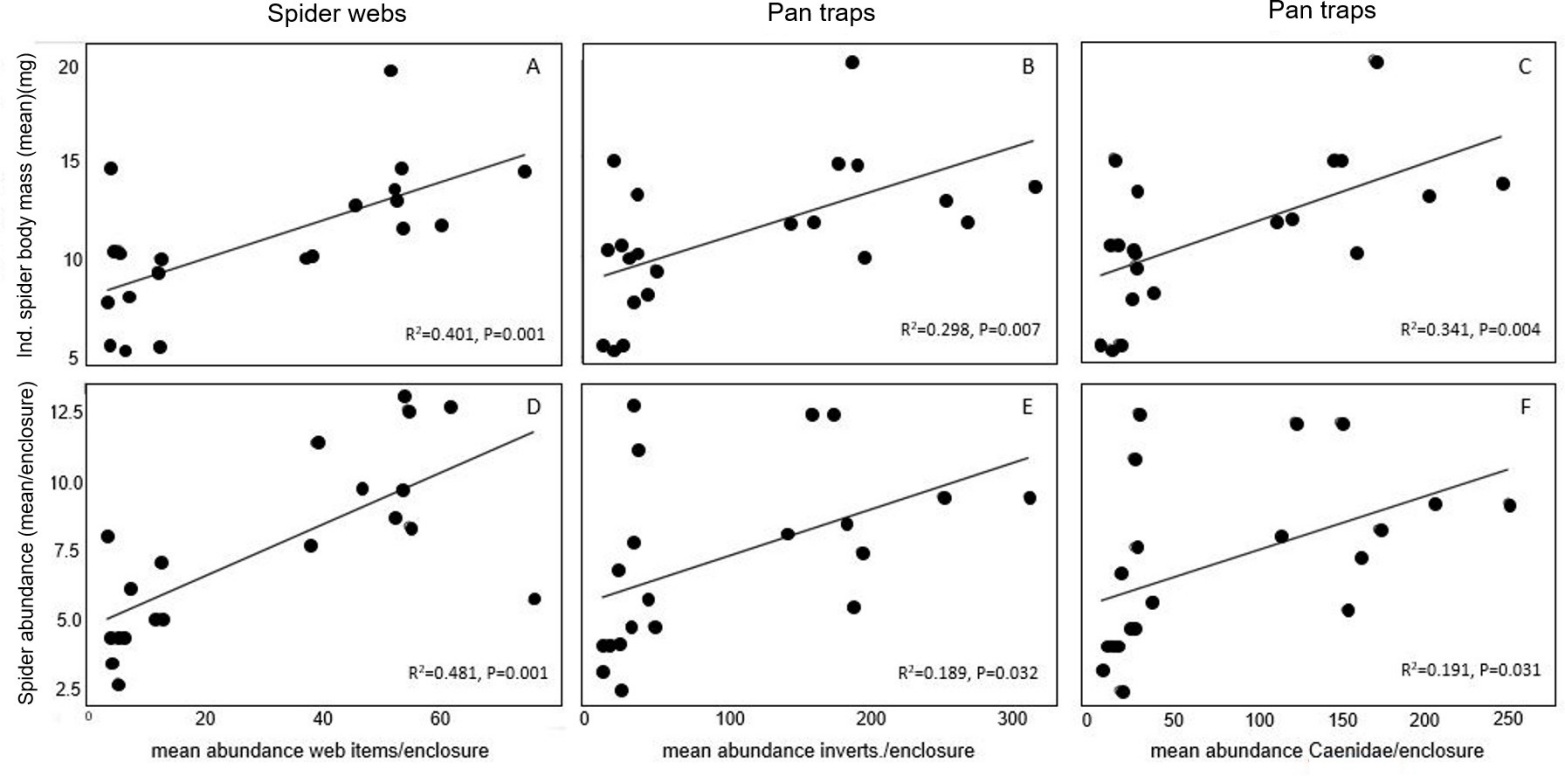
Relationships between spider attributes and insect communities in pan traps and in webs. Positive linear relationships were observed between spider body mass and: number of prey items in webs (A), number of invertebrates caught in pan traps (B), and number of Caenidae mayflies caught in pan traps (C). Relationships were also found between spider abundance and the number of prey items in webs (D), number of invertebrates caught in pan traps (E), and number of Caenidae mayflies caught in pan traps (F).

Linear regressions revealed positive relationships between spider body mass and the number of prey items in webs (R^2^ = 0.40, p = 0.001) (Fig 3A), the number of emergent and terrestrial invertebrates collected in pan traps (R^2^ = 0.298, p = 0.007) (Fig 3B), and the number of Caenidae mayflies caught in pan traps (R^2^ = 0.341, p = 0.004) (Fig 3C). Spider abundance was positively related to prey items in webs, emergent and terrestrial invertebrate abundance, and Caenidae abundance (R^2^ = 0.481, p = 0.001; R^2^ = 0.189, p = 0.032; R^2^ = 0.191, p = 0.031) (Fig 3D–3F). Caenidae was by far the most abundant family of emergent invertebrates in pan traps.

### Spider web prey abundance

**Fig 4:**
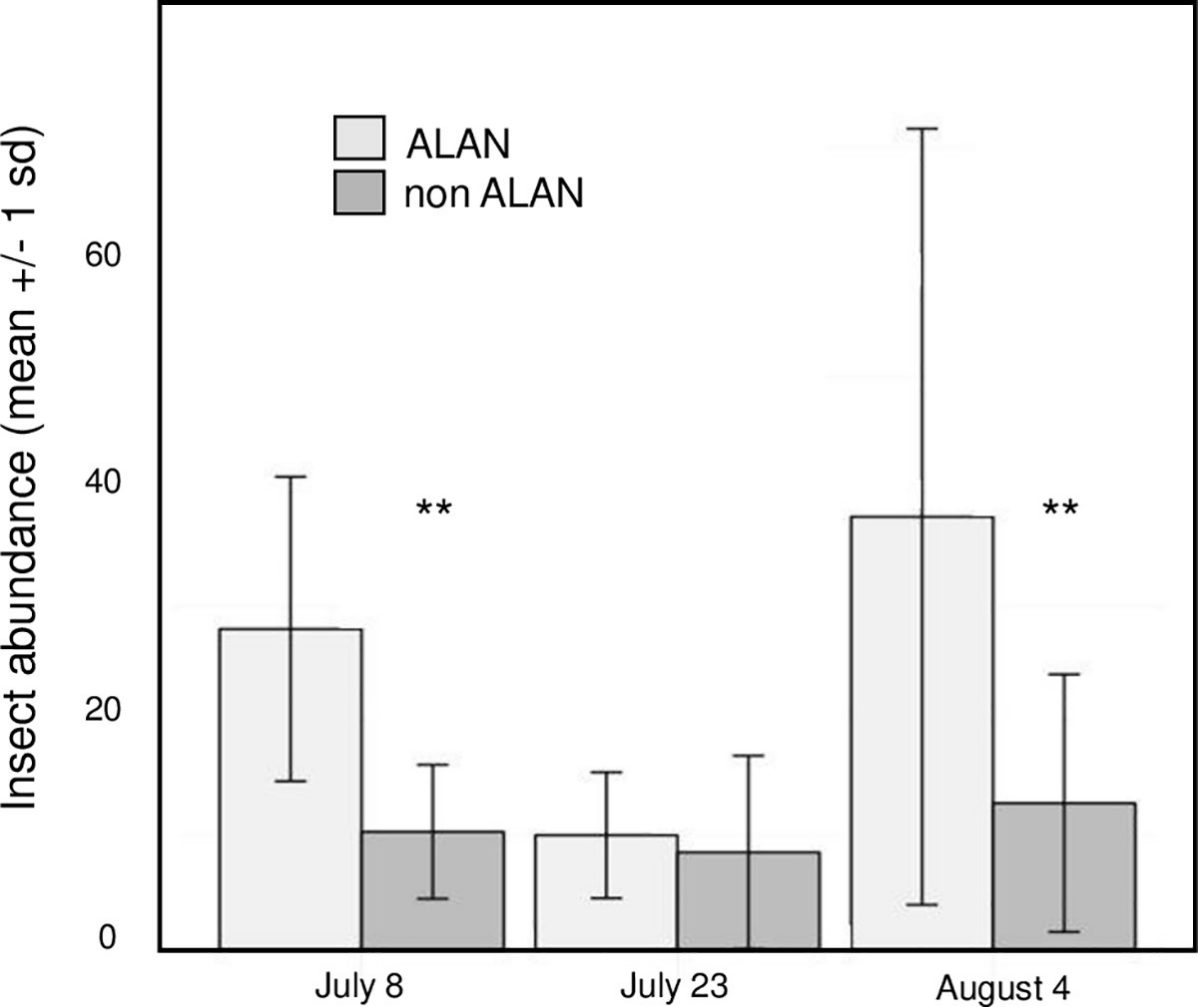
Insect-prey abundance captured in webs. Across all sampling dates the mean number of insects captured in spider webs was greater in the presence of ALAN relative to controls, an effect that depended on sampling date.

Across all sampling dates the number of prey items captured in spider webs was on average 139% greater on ALAN mesocosms than those built on no-ALAN mesocosms (rmANOVA, F_1,18_ = 15.13, p<0.001) (Fig 4). There was no significant interaction between the light treatments and sampling date (rmANOVA, F_2,36_ = 2.50, p = 0.095). On the first sampling date the number of prey items in webs was 172% greater in ALAN mesocosms than control mesocosms then dropped to an 18% difference in abundance on the second sampling date. The third sampling date showed webs build on ALAN mesocosms had 195% greater abundance of prey items than control mesocosms.

### Invertebrate community

**Fig 5:**
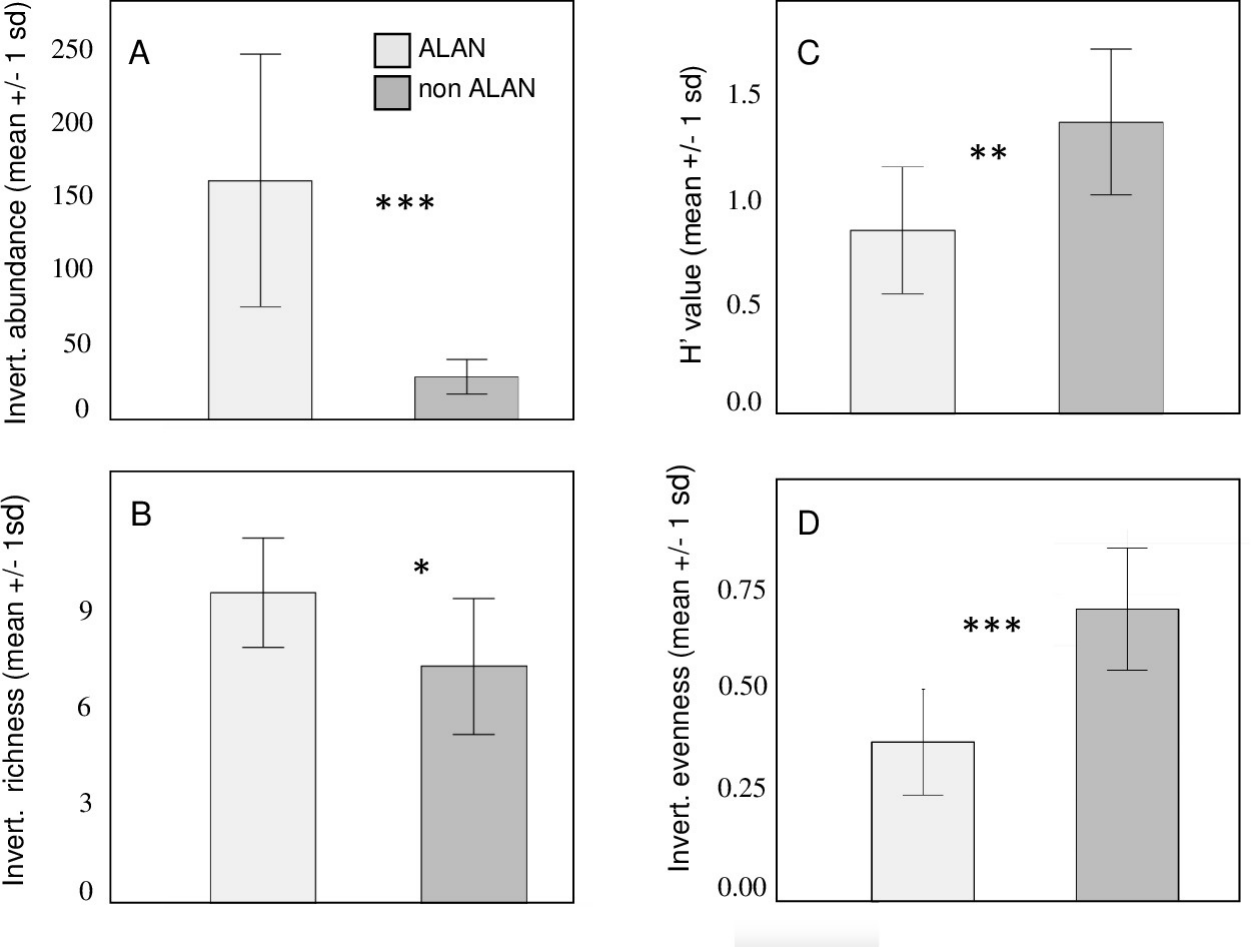
Terrestrial and emergent invertebrate community attributes, sampled via pan traps. ALAN treatments had significantly more invertebrates that non-ALAN treatments (Fig 5A) and had greater taxonomic richness (Fig 5B). Species diversity was greater in non-ALAN treatments (Fig 5C) and non-ALAN treatments had more-even invertebrate communities (Fig 5D).

Invertebrate abundance, richness, diversity, and evenness in pan traps were all significantly influenced by ALAN. One thousand nine hundred and ninety individual invertebrates representing thirty-six emergent insect and terrestrial invertebrate families were collected from pan traps. Invertebrate abundance was on average 456% greater in ALAN plots than in non-ALAN plots (ANOVA, F_1,18_ = 23.6, P<0.001) (Fig 5A); ALAN plots had on average 31% greater taxonomic richness than controls (ANOVA, F_1.18_ = 7.16, P = 0.015) (Fig 5B). Control mesocosms, however, had species diversity values (H’) that were 58% greater than ALAN mesocosms (ANOVA, F_1,18_ = 12.5, P = 0.002) with mean values (+/- SD) of 1.398 (+/- 0.35) and 0.879 (+/- .30) respectively (Fig 5C). Control mesocosms also had 83% more-even invertebrate communities than ALAN mesocosms (ANOVA, F_1,18_ = 27.0, P<0.001) (Fig 5D). Differences in H’ and evenness can be attributed to Family Caenidae, which was the most abundant family across all treatments and which was 818% more abundant in ALAN treatments than non-ALAN treatments (ANOVA, F_1,18_ = 20.0, P<0.001).

**Fig 6:**
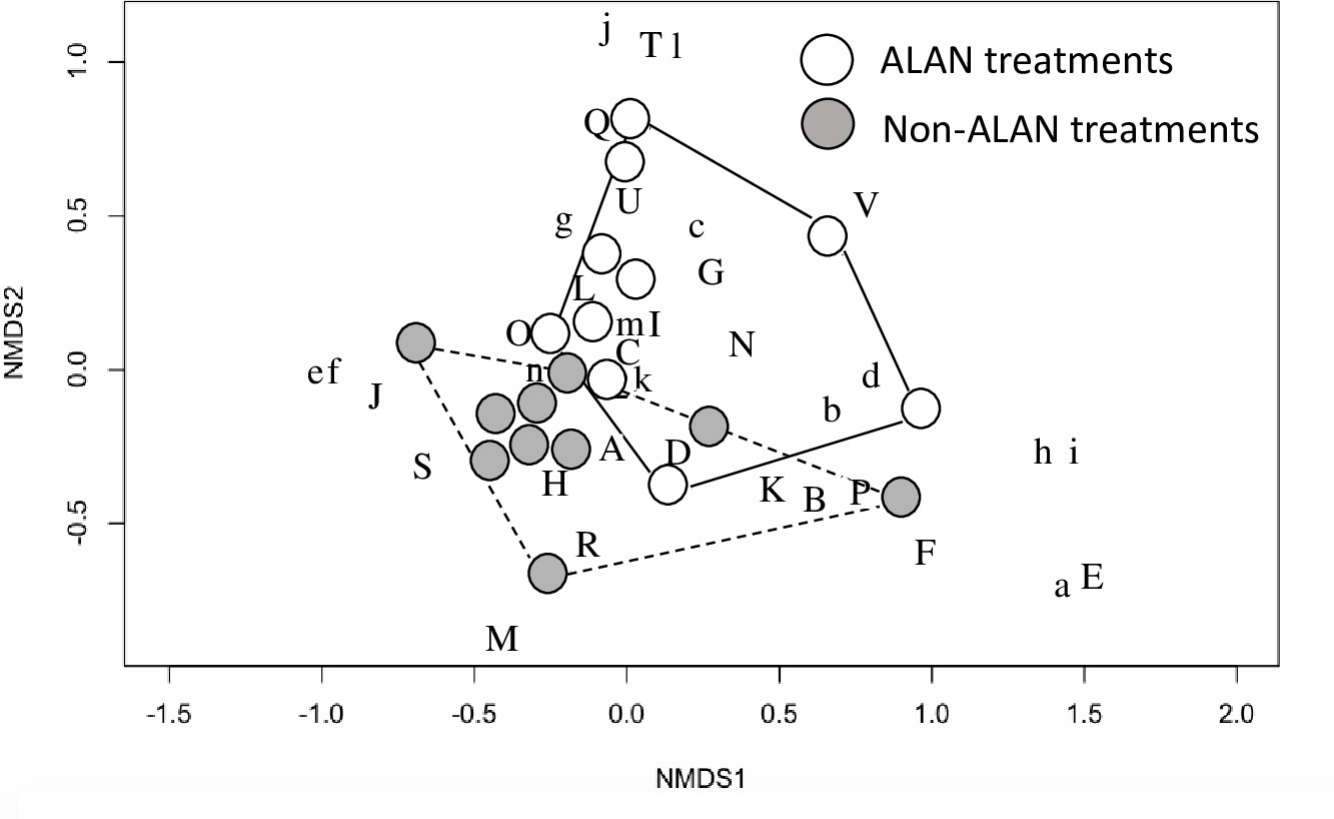
NMDS ordination plot showing community composition of terrestrial and emergent invertebrate communities from pan trap samples. Closed circles represent ALAN mesocosms and open circles represent no-ALAN mesocosms. MRPP indicated significant difference between lit and unlit communities (P = 0.002). Capital letters represent emergent invertebrate families, while lowercase letters represent terrestrial invertebrate families. Invertebrate families are indicated by letters as the following: (A) Caenidae, (B) Hydropsychidae, (C) Chironomidae, (D) Ceratopogonidae, (E) Tabanidae, (F) Sciomyzidae, (G) Baetidae, (H) Culicidae, (I) Ephydridae, (J) Corixidae, (K) Empididae, (L) Notonectidae, (M) Limnephilidae, (N) Tipulidae, (O) Haliplidae, (P) Coenagrionidae, (Q) Hydroptilidae, (R) Helodidae, (S) Hydrophilidae, (T) Phryganeidae, (U) Polycentropodidae, (V) Leptoceridae, (a) Cosmopterygidae, (b) Aphidae, (c) Dolichopodidae, (d) Tetragnathidae, (e) Muscidae, (f) Delphacidae, (g) Chrysomelidae, (h) Agromyzidae, (i) Mymaridae, (j) Araneidae, (k) Formicidae, (l) Cicadellidae, (m) Noctuidae, (n) Acrididae.

Invertebrate-community composition from pan-trap samples was significantly different in ALAN mesocosms than in controls, as shown by NMDS (MRPP, P = 0.002, A = 0.157) (Fig 6). ALAN and non-ALAN plots separated along NMDS axis 2. End members of this gradient include Araneidae (j), Phryganeidae (T) and Notonectidae (L), which were associated will ALAN communities; Limnephilidae (M), Cosmopterygidae (a), Muscidae (e), and Sciomyzidae (F), which were associated with non-ALAN communities. The number of terrestrial invertebrates captured did not differ between ALAN and non-ALAN treatments (ANOVA, F_1,18_ = 2.24, P = 0.152), but the number of emergent insects was significantly greater in ALAN treatments (ANOVA, F_1,18_ = 26.2, P<0.001).

## Discussion

Resource exchanges are an intrinsic facet of riparian and aquatic ecosystems that contribute to their biodiversity and productivity [2, 44], and these exchanges are sensitive to human activities [45]. Research to date has focused on resource subsidies from terrestrial to aquatic ecosystems. Here we detail some ways in which anthropogenically altered lighting can influence the flow of resources in the other direction—from aquatic to terrestrial ecosystems in the form of emergent aquatic insects. A key distinction between our study and others is that ALAN was positioned directly over the water, a design choice that closely mimics dock lighting, and other types of lighting that are common at the terrestrial-aquatic interface. This created localized patches of horizontally polarized light reflecting from the water’s surface; this is a possible reason for the large number of aquatic insects that we observed in our ALAN plots. Additionally, ALAN altered the attributes of insect communities, affecting overall abundance, diversity, richness and evenness. Aquatic insects are preferred by spiders as a food source over their terrestrial counterparts [4, 14], and we observed elevated spider abundance and body mass under ALAN exposure. Interestingly, body mass increased dramatically in female spiders relative to their male counterparts. In aggregate these results show how ALAN can impact fluxes of resources between aquatic and terrestrial ecosystem, the consumers of these resources, and how the effects can be sex-dependent.

We found a strong increase in insect abundance in ALAN plots, with these communities dominated by emergent aquatic insects, a finding that is consistent with those from other studies [28, 32, 34, 37, 46, 47]. ALAN plots also had greater family richness, while non-ALAN control plots were more diverse, a result attributable to more-even community composition; ALAN mesocosms were dominated by the aquatic family Caenidae, reducing evenness and H’ values. Members of Ephemeroptera, Trichoptera, and Diptera are highly attracted to horizontally polarized light and use this reflected light to find suitable habitat for ovipositioning [48–50]. Our placement of the lighting immediately over the water–a choice that mimics dock lighting and other light sources that are in close proximity to water–may have facilitated the increase in insect abundance we observed, with a potential consequence being reduced fluxes of emergent insects into the riparian zone. NMDS found that some families were strongly associated with ALAN and non-ALAN treatments. For example, Araneidae, Phryganeidae, and Notonectidae, which were associated will ALAN; Limnephilidae, Cosmopterygidae, Muscidae, and Sciomyzidae, which were associated with the non-ALAN treatment. These families may prove to be useful as bioindicators of the effects ALAN has on invertebrate communities.

Two conceptual models–the captivity effect and the vacuum-cleaner effect [51]–have relevance for the results we observed. The captivity effect refers to the capture of insects at a light source or their fixation on a light source, trapping them in that lit area. The vacuum-cleaner effect refers to artificial light attracting insects from unlit areas into lit areas. Substantially more emergent insects were sampled in ALAN plots. Although our experimental design does not allow us to distinguish the degree to which this result is due to the vacuum-cleaner or the captivity effect, we suspect that the vacuum-cleaner effect was more pronounced given 1) the presence of terrestrial invertebrates in the plots and 2) the large number of invertebrates that were sometimes sampled, which would seemingly be beyond the productivity of our small one by one meter plots. The overall effect is evidenced by our NMDS analysis where clear differentiation was observed between ALAN and non-ALAN treatments, with many emergent insect taxa, such as Caenidae, clustered with ALAN treatments indicating a greater attraction to light. In several studies where light sources were placed along the shore, emergent aquatic insects were attracted away from the water towards ALAN, illustrating the vacuum-cleaner effect [32, 34, 37]. The location of the lighting appears to determine where fluxes of emergent insects will accumulate. Physiological and life-history differences between invertebrate species may support why we saw an ALAN on emergent aquatic invertebrates, both drawn to and captured by light, but not a vacuum-cleaner effect on terrestrial invertebrates. Further examination of the interaction between light source, reflective surface, characterization of the light field and the orientation of the light source on a surface would seem to be a fruitful area for future research, as well as how insect taxa react to different stimuli.

In addition to seeing an increased abundance of emergent insects at ALAN plots, we also observed concomitant increases in the abundance and body mass of tetragnathid spiders. Because these spiders can track prey via a numerical response [5, 15], and depend heavily on resource subsidies from aquatic environments [15, 16], the abundance of spiders in natural areas has the potential to shift toward artificially lit areas where invertebrate prey is greatest. Tetragnathids are interception predators and can disrupt the flow of resources across ecosystem boundaries through consumption or capture of prey [52]. Support for this is seen in carbon isotope analysis, revealing that 40–60% of riparian spider diet can be composed of aquatic sources [4, 14]. These and other studies highlight how spiders can facilitate exchange of resources from aquatic to terrestrial ecosystems by concentrating aquatic resources in riparian zones that may have otherwise dispersed elsewhere. It remains unclear if ALAN at the aquatic-terrestrial interface results solely in a redistribution of tetragnathid spiders, or in increased spider populations, through the interception of resources that would have otherwise dispersed across the broader landscape.

The greater abundance and body mass of tetragnathids in ALAN plots, combined with the much greater abundance of emergent insects there, illustrates the effect ALAN can have on the movement of subsidies from aquatic to terrestrial ecosystems. As spiders move into artificially lit areas and away from those of natural darkness, they may alter the flow of aquatic resources with potential cascading consequences in coupled aquatic-riparian environments. Manfrin et al. (2017) observed that subsidy exchange became more limited outside of an artificially lit area, and aquatic-derived energy shifts have been observed on an ecosystem level in stream/riparian coupled systems [53]. In our study, ALAN was positioned immediately over the water and the spiders were found to be highly abundant on emergent aquatic vegetation. ALAN and the elevated spider abundance that resulted likely prevented emergent insect resources from entering the riparian zone, but rather, concentrated them in the littoral zone. These resources were likely then cycled within the lake. The lack of a response by terrestrial insects demonstrates that the effects of ALAN on exchanges between aquatic and terrestrial ecosystems can be asymmetrical, having a greater impact through ‘vacuum cleaning’ and ‘captivity’ on emergent aquatic insects rather than terrestrial insect inputs.

Like most areas in North America, our study sites experience skyglow from surrounding human development, and our ALAN additions were therefore not done in conditions of natural darkness. Rather, our experiment augmented existing ALAN, simulating the common anthropogenic practice of adding light to areas that are experiencing increasing development. Had our experiment been done under darker conditions our experimental ALAN additions may have been more conspicuous and attracted a greater number of insects. Alternately, the long-term presence of skyglow could have altered light-sensitive members of the invertebrate community, and altered our results. Productive areas of future research are to evaluate the iterative addition of ALAN to human landscapes to simulate future light regimes as development and light intensity increase across the globe.

Our study made use of a replicated field experiment, and the design was constrained by limited amount of littoral area and appropriate abiotic conditions, such as water depth. Light from ALAN treatments illuminated adjacent plots and this ‘cross-talk’, along with skyglow and moonlight, can explain the fairly high light measurements that were taken from non-ALAN treatments. Viewed from a distance, the aggregation of our ALAN plots in a single littoral area of the lake may have made them more conspicuous to invertebrates, and may have resulted in a stronger vacuum-cleaner effect than if the mesocosms had been more dispersed. Despite these challenges the addition of experimental ALAN was well within the range of ALAN found in the literature, both in the context of experimentally added ALAN, and that observed in human-inhabited landscapes.

Responses by predators to ALAN have been inconsistent across studies. Most have documented increases in predator abundance or body mass in response to ALAN [31, 36, 37], but others observed reduced abundance or fitness traits. Several studies have shown ALAN confers benefits to invertebrate predators such as terrestrial beetles and spiders [31, 34, 54]. A decline was observed, however, in the fitness of non-tetragnathid orb-weaver species in the presence of ALAN [55, 56]. Bats have been observed both taking advantage of ALAN [57], as well as avoiding artificially lit areas [58]. Predation rates of a wasp species increased under low levels of ALAN but decreased under high levels of ALAN [59]. When taken together, these results show the intensity of ALAN potentially plays a role in predator abundance and fitness and that role changes based on the taxonomic identity of both prey and predator.

We found that the effect of ALAN on spider size was strongly sex-dependent, with females responding substantially more than males. Tetragnathids are sexually dimorphic and females are larger than males, and in light of this, it isn’t surprising that females accrued more absolute biomass than males. However, even when adjusting increases in mass by expressing results on a percent-mass basis, female body mass still increased by 176% in ALAN plots relative to controls; body mass of males increased by only 34%. While the mechanisms behind this finding are not clear, it suggests that females are more-strongly food-limited than males, and have the ability to rapidly increase in mass in the presence of elevated food availability. A recent study found that gravid tetragnathid spiders increased prey capture and consumption compared to non-egg laying females of the same species [60], suggesting that ALAN can alleviate carbon limitation during egg development. An alternate but not exclusive explanation is that large-bodied spiders were able to occupy the preferred feeding habitat that ALAN provided, supplanting smaller individuals. The consequences of ALAN for reproductive output and fitness between sexes would seem to be fruitful areas for future research.

A large body of research has shown that resource subsidies are an integral dimension of ecosystem functioning and productivity [e.g., 2, 61, 62], but we are only beginning to understand how human activities alter flows of resources across ecosystem boundaries. Freshwaters and their riparian zones are among the most-impacted ecosystems on the planet, with impacts to littoral, shoreline and riparian habitats stemming from multiple human stressors [44]. Similarly, aquatic and terrestrial insects are in states of decline with 41% of insect species in decline and 33% of aquatic insects threatened [63]. Here we showed how ALAN at the aquatic-terrestrial interface impacts fluxes of aquatic insects, and that the response by riparian consumers can be pronounced and sex-dependent. When ALAN is located in close proximity to freshwater it can be expected to concentrate fluxes of emergent aquatic insects, especially when polarized light is reflected from the surface of the water. Our findings also show terrestrial predators in the littoral zone can compound this effect and intercept resource flows and prevent them from entering the terrestrial realm.
